# Intracranial and Intraocular Pressure at the Lamina Cribrosa: Gradient Effects

**DOI:** 10.1007/s11910-018-0831-9

**Published:** 2018-04-12

**Authors:** Gauti Jóhannesson, Anders Eklund, Christina Lindén

**Affiliations:** 1 Department of Clinical Sciences, Ophthalmology, Umeå University, Umeå, Sweden; 2 Wallenberg Centre for Molecular Medicine, Umeå University, Umeå, Sweden; 3 Department of Radiation Sciences, Biomedical Engineering, Umeå University, Umeå, Sweden

**Keywords:** Translamina cribrosa pressure difference, Translaminar pressure difference, Cerebrospinal fluid, Intracranial pressure, Intraocular pressure, Normal tension

## Abstract

**Purpose of Review:**

A pressure difference between the intraocular and intracranial compartments at the site of the lamina cribrosa has been hypothesized to have a pathophysiological role in several optic nerve head diseases. This paper reviews the current literature on the translamina cribrosa pressure difference (TLCPD), the associated pressure gradient, and its potential pathophysiological role, as well as the methodology to assess TLCPD.

**Recent Findings:**

For normal-tension glaucoma (NTG), initial studies indicated low intracranial pressure (ICP) while recent findings indicate that a reduced ICP is not mandatory.

**Summary:**

Data from studies on the elevated TLCPD as a pathophysiological factor of NTG are equivocal. From the identification of potential postural effects on the cerebrospinal fluid (CSF) communication between the intracranial and retrolaminar space, we hypothesize that the missing link could be a dysfunction of an occlusion mechanism of the optic nerve sheath around the optic nerve. In upright posture, this could cause an elevated TLCPD even with normal ICP and we suggest that this should be investigated as a pathophysiological component in NTG patients.

## Introduction

The importance of an imbalance between intracranial pressure (ICP) and intraocular pressure (IOP) in the pathophysiology of several diseases involving the eye and the brain has received increased attention in recent years [1, 2•, 3]. It has long been known that elevated ICP causes papilledema and the so-called “choked disc” that eventually results in axonal death and visual loss [4]. Observations of visual disturbances with disc swelling and increased ICP in astronauts and the subsequent presentation of the spaceflight-associated neuro-ocular syndrome (SANS) have created increased interest in this relationship.

The idea of detrimental pressure difference, between the eye and the brain as part of glaucoma pathophysiology, is not new. It was first described as a possible contributing cause of glaucoma in the 1970s [5, 6]. Recently published studies on glaucoma [7, 8•, 9] have further brought the hypothesis that abnormal TLCPD is a contributing factor in glaucoma pathophysiology, to the fore.

In this review, we aim to summarize what is known from publications about the translamina cribrosa pressure difference (TLCPD) and the related pressure gradient effect over the lamina cribrosa (LC), and its potential as a pathophysiologic component in optic nerve head (ONH) diseases.

## Translamina Cribrosa Pressure Difference and Gradient Effects

Gradient effects from differences between ICP and IOP are found at the ONH, specifically at the LC. The LC is a trabecular structure of several layers and pores of different sizes through which the optic nerve fiber bundles pass, and it is a continuation of the inner layer of the posterior sclera. IOP is the pressure within the intraocular compartment anteriorly of LC. Posteriorly, the optic nerve (ON) is surrounded by the three layers of meninges: dura mater, arachnoid mater, and pia mater. The cerebrospinal fluid (CSF) filled subarachnoid space ends within a blind pouch behind the LC [2•, 10, 11]. Since the subarachnoid space of the intraorbital optic nerve is continuous with the subarachnoid space surrounding the brain and spinal cord, it has been assumed that the ICP of the brain is evenly transferred all the way to the posterior part of the LC. The LC separates the ocular cavity with IOP from the retrolaminar subarachnoid pouch with ICP. Accordingly, TLCPD is defined as IOP–ICP and the difference at the level of LC causes a pressure gradient effect. A gradient is a difference in pressure per unit distance and therefore LC thickness needs to be known to be able to calculate the translaminar gradient. However, LC thickness is not known in human in vivo studies and thus TLCPD is used as a surrogate.

## Pathophysiologic Theories—Importance of TLCPD

Volkov [6] suggested that ICP was of importance in the pathophysiology of glaucoma. Since then, disturbed TLCPD has been put forward as an important factor in the pathogenesis of diseases such as SANS and glaucoma.

The basic hypothesis has been that increased TLCPD is detrimental to the axons of the optic nerve via a mechanical insult and/or through a disturbed axoplasmic transport, which then causes edema. A change in either IOP or ICP may affect the homeostasis of the ONH. The imbalance can be due to increased ICP, e.g., in SANS and idiopathic intracranial hypertension (IIH), or to elevated IOP as in the case of glaucoma. It is, however, important to remember that there are large individual differences in the biomechanical properties of the LC that determine how susceptible the axons are to this stress [3, 12]. This has been shown in a monkey model where IOP and ICP have been controlled and LC deformation measured [12].

In the last decade, it has also been postulated that possible low ICP in normal-tension glaucoma (NTG) patients might contribute to NTG pathophysiology through increased TLCPD [13]. Morgan et al. (2016) has discussed the possible influence of orbital pressure on the pressure in the optic nerve subarachnoid space (ONSAS) and that it might buffer large TLCPD effects when ICP is very low. They hypothesized that low orbital pressure and decreased elasticity of the pia mater could lead to increased transfer of low pressures from the orbit and the ONSAS to the retrolaminar optic nerve [14•]. Additionally, the thickness of LC in enucleated eyes has been shown to decrease with advancing glaucoma [15] and since the pressure gradient is dependent on the thickness of LC, an eventual gradient due to TLCPD will be greater with a thinner LC [16].

It has been shown that ICP decreases with age [17, 18]. Wostyn et al. [19] discussed that perhaps it is not the low ICP per se that is problematic but rather a decreased CSF production and turnover, as demonstrated in diseases such as Alzheimer [20]. They suggested an alternative explanation for NTG development—the low ICP may be due to CSF circulatory failure which causes disturbed neurotoxin clearance along the optic nerve [19]. This has been supported by findings of lower CSF flow-range ratio in the ONSAS of NTG patients [21]. Furthermore, a hypothesis that high ICP fluctuations, i.e., rhythmic oscillations in ICP, may be an independent risk factor for glaucoma has also been presented [22].

An interesting new concept is the postulated presence of a perivascular transport system for waste clearance in the eye and the brain, i.e., the “glymphatic system” which is another means to move extracellular fluid [23–25]. A reason why high TLCPD could be detrimental to the axons of the optic nerve could therefore be that it causes a restriction of normal glymphatic flow, leading to accumulation of toxic substances around axons and consequently damage to the axons of the optic nerve [26].

## Experimental Studies on TLCPD

Measurement of the LC with high enough resolution to estimate its thickness with acceptable accuracy is difficult in human in vivo studies. Furthermore, direct measurement of the retrolaminar pressure in humans is not possible due to practical and ethical considerations. Thus, the evaluation of gradient effects and their potential detrimental effect on the ONH is challenging. These obstacles can to a certain extent be overcome by using animal models.

In a study on dogs, Morgan et al. studied the gradient over the LC. A micropipette linked to a servonull pressure system was moved through the LC and into the optic nerve subarachnoid space (ONSAS). IOP and ICP were monitored continuously, and the gradient over LC was measured at various IOP and ICP levels. They found that there was a strong correlation between the gradient and TLCPD when ICP was higher than 0 mmHg [27]. In another study on dogs, Hou et al. demonstrated that ICP and retrolaminar pressure were positively correlated in a specific pressure range but when ICP was lowered below a critical point of 3 mmHg, TLCPD stabilized due to a constant retrolaminar pressure [28]. The results from these dog models showed that ICP and retrolaminar pressure were generally in good agreement and thus it is possible to use ICP in the calculation of TLCPD. That agreement persists until ICP falls below a certain level, indicating that the communication and pressure transfer between ONSAS and the rest of the intracranial CSF system is lost at low ICP levels.

Yang et al. studied the effects of chronically lowered ICP in a monkey model. Compared to controls, they showed that low ICP caused optic neuropathy while IOP remained stable within normal range, supporting the hypothesis that alternation on one side of the LC is sufficient to cause disturbed balance between IOP and ICP and may cause a harmful effect on the ONH [29]. In an experimental setup with porcine eyes, phase-contrast micro-computed tomography was used to investigate the effect of different ICP levels on LC and retrolaminar tissue while IOP was kept stable. Changes in ICP were found to cause significant deformation on both LC and the retrolaminar tissue [30]. Skrzypecki et al. investigated the effect of high blood pressure and posture on TLCPD with hypertensive rats compared with wild type. In the upright position, ICP in the ventricles was found to decrease significantly while IOP did not change, which resulted in increased TLCPD in both groups. No difference was found between the groups. The same relationship between IOP, ICP, and postural changes has also been shown in rabbits [31] and cats [32]. Thus, these studies confirm that posture has different effects on IOP and ICP [33].

Morgan et al. [34] showed in a dog model with confocal scanning laser tomography that a displacement of the disc surface occurred mostly when the TLCPD was low and that little extra movement was measured when TLCPD was > 15 mmHg. Studying LC with OCT, Wang et al. [12] recently investigated the IOP and ICP effect on LC in monkeys. The study showed that LC microstructure was deformed as a response to acute alternations in both IOP and ICP. Decrease in LC pores and thickening of LC beams were seen with increased IOP or ICP. However, when both pressures where high, the LC pores increased. Thus, the authors emphasized the importance of considering both IOP and ICP in ONH assessment [12].

Taken together, these animal studies show that there is a relationship between effects on the LC and TLCPD, particularly if ICP is low. Furthermore, there is an indication of a protective mechanism against too low retrolaminar pressure in cases of negative ICP.

## Measurement of Intracranial and Intraocular Pressure

The approach to investigate TLCPD is to assess the pressure difference between the anterior and posterior side of the LC. In clinical settings, these parameters are not trivial to measure.

### Intracranial Pressure

For the posterior side, the challenges are much greater than for the anterior side and require some critical assumptions. In published clinical studies on TLCPD, assessment of retrolaminar pressure is based on invasive CSF space accessed through lumbar puncture and the assumption of a communicating CSF system from the lumbar space through the intracranial compartment to the subarachnoid space retrolaminarily. In clinical routine, ICP is commonly measured invasively through direct lumbar puncture and has been shown to agree well with pressure measured intracranially [35, 36]. Due to the invasive nature and the risk for complications, several non-invasive methods have been developed although none has yet been proven clinically reliable [37–40].

### Intraocular Pressure

Regarding the anterior side, IOP measured indirectly trans corneally must be considered a reasonable substitute, especially if corrected for hydrostatic distance to LC. In clinical practice, the measurement of IOP is always an indirect measurement, i.e., an estimation of the true pressure inside the eye, and all tonometry methods suffer from sources of error [41–43]. The most widely used method is Goldmann Applanation Tonometry (GAT) [44]. Biomicroscope mounted GAT and many other methods are limited to use with the patient in a sitting position. Handheld techniques that are not limited to upright position exist. These include, e.g., Icare^®^ [45], which can measure IOP in both supine and sitting positions and Applanation Resonance Tonometry^®^ [46, 47], which can measure IOP regardless of body position.

## Clinical Implications of TLCPD

In this section, we will first briefly discuss findings related to IIH and SANS, which are conditions related to reduced TLCPD. We will then put the main focus on the importance of TLCPD in glaucoma with a suspected elevated TLCPD.

### Idiopathic Intracranial Hypertension (IIH)

Classic signs and symptoms of intracranial hypertension are optic disc edema, headaches, pulsatile tinnitus, transient visual obscurations, and radicular pain. Some patients also demonstrate choroidal folds and hyperopic shift. IIH affects mostly obese females of child-bearing age, and reasons for this remain poorly understood. The disease can cause visual field defects similar to glaucoma, i.e., nerve fiber bundle defects, and can potentially cause blindness [48]. For the IIH, the elevated ICP is, as the name suggests, a part of the definition. Thus, the relationship between IOP and ICP may play a role in IIH patients. With increased ICP and normal IOP, the reduced or even reversed TLCPD causes anterograd gradient effects towards the eye. In a study with ocular coherence tomography (OCT), patients with normal IOP and papilledema due to elevated ICP were divided into two groups with high TLCPD and low TLCPD. As the papilledema resolved, the LC in patients with high TLCPD was found to move backward, which was not the case when TLCPD was low. Furthermore, a significantly larger retinal nerve fiber layer thinning was found in the high TLCPD group. Since LC movement was not found when TLCPD was low, the authors speculate that LC position could be used as surrogate for TLCPD in patients with intracranial hypertension and normal IOP [49].

Another examples of the importance of ICP/IOP relationship, and not only absolute ICP values, in patients with IIH can be seen in reports on glaucoma patients who have developed papilloedema after successful IOP-lowering surgery with normal postoperative IOP levels. Further work up revealed that the patients also had IIH [50–52]. These findings suggest that IOP has a role in IIH and that increased IOP may indeed be a protective factor in IIH patients [13].

### Spaceflight-Associated Neuro-ocular Syndrome (SANS)

During the last decade, it has become apparent that astronauts traveling in space are at risk to acquire ocular abnormalities such as optic disc edema, choroidal folds, and hyperopic shift. This disease has similar ocular characteristics as found in IIH patients but the symptomatology differs since IIH symptoms of headaches, tinnitus, and transient visual obscurations are not common in SANS patients [53]. However, the clinical similarity together with potential venous stasis due to cephalad fluid shifts in microgravity has led to the suspicion of elevated ICP in the astronauts. The condition was previously called visual impairment/intracranial pressure syndrome (VIIP) but the syndrome has recently been redefined by National Aeronautic and Space Administration (NASA) as SANS [54]. The condition has been identified as one of the major risks in human space exploration [55]. In a study by Mader et al., seven astronauts who had been in space for approximately 6 months were reported to have visual disturbances. Post-flight LP-investigation of four of them revealed increased ICP in addition to the above-mentioned ocular findings [53]. This underlined the significance of TLCPD as a possible pathophysiological component and that more research is needed. Kramer et al. further studied astronauts returning from the International space station (ISS) and confirmed SANS findings in most of the subjects [56]. IOP has been assessed at the ISS while ICP is currently under investigation with non-invasive methods. In a study showing the gravitational postural dependence for ICP, Eklund et al. suggested that even without an elevation of ICP, the 24-h mean ICP can become higher in microgravity than on earth [57•], since the normal reduction to around zero pressure in upright posture is due to gravity. This was supported by measurements in microgravity conditions in parabolic flights that showed a non-pathologically elevated ICP [58].

### Glaucoma

The hypothesis that glaucoma is caused by an increased translamina cribrosa pressure gradient has led to the assumption that particularly normal-tension glaucoma (NTG) may be caused by a low ICP, which would create a similar mechanical condition as an elevated IOP [1, 2•, 8•, 9, 13, 14•, 59].

Numerous articles have been written on the subject. However, if the literature is critically evaluated, there are limited scientific studies that investigate this hypothesis. Most of the publications are reviews [1, 2•, 60–66], case reports [67–69], or indirect measurements of ICP [70–76].

There are several controversies in the literature regarding TLCPD. One concerns the methods used to measure ICP. As stated before, invasive direct measurement of ICP is the golden standard in clinical practice. A number of different indirect methods to estimate or indicate ICP have been described. Although non-invasive methods would be preferable due to the risk of complications with the invasive ones, the indirect methods are not considered to be sufficiently reliable for clinical practice [37–40]. Still, conclusions on TLCPD are based on studies that used indirect methods to estimate TLCPD [70, 71, 73, 74, 76].

Starting with the non-invasive ICP approaches, it has been shown that the width of the optic nerve subarachnoid space (ONSASW) estimated with magnetic resonance imaging (MRI) is positively correlated to ICP measured with lumbar puncture (LP) [77]. Wang et al. (2012) studied ONSASW in NTG, high-tension open angle glaucoma (HTG), and non-glaucomatous controls in a prospective observational study in a Chinese population. Overall, they found narrower ONSASW in NTG than in HTG and controls, which indicates lower ICP in NTG patients [76]. Similarly, but by using ultrasound, Liu et al. recently showed that the area of the subarachnoid space of the optic nerve was smaller in NTG than in healthy controls [78]. Jonas et al. [73] used a formula based on body mass index (BMI), blood pressure, and age [77] to estimate ICP and TLCPD in participants in the Beijing Eye Study. These calculated ICPs were lower in the patients with glaucoma than in non-glaucomatous participants [73]. One interpretation of this could be that glaucoma risk increases with age, low blood pressure, and low BMI. Similarly, Lee et al. used the same mathematical formula to estimate ICP. They found an association between higher TLCPD and NTG with high-teen IOP values, but the same was not true for low-teen NTG patients [74].

Contrary to these findings, both Jaggi et al. and Pircher et al. used computerized tomography (CT) to measure optic nerve sheath diameter (ONSD) in NTG compared with controls and found significantly increased diameter in NTG patients. Pircher et al. also compared ONSD to ICP measured with LP in NTG patients and found no correlation between the parameters. Their results can be interpreted as speaking against the hypothesis of NTG patients having low ICP, but the authors discussed other possible explanations for their findings, e.g., compartmentation [71, 79]. Furthermore, Pinto et al. used sonography to measure the diameter of the optic nerve sheath in healthy controls, NTG, and HTG and found no significant difference [70].

Another indirect method to estimate ICP is the two-depth transorbital Doppler technique. It uses ophthalmic artery as an intracranial pressure sensor [80]. The method has been validated, and although not ready for use in clinical practice, it has shown promising agreement when compared to neurological patients undergoing LP [39, 81]. In a study with transorbital Doppler non-invasive ICP on NTG, HTG, and healthy controls (*n* = 9/group), they found a non-significant tendency towards reduced ICP and a higher TLCPD in glaucoma patients. Note that the glaucoma patients had not undergone washout of IOP-lowering treatment and that IOP was measured in the sitting position, while ICP was measured in the supine position [75].

In summary, studies that use ONSASW, BMI, blood pressure, age, ophthalmic blood flow profile, and other indirect indications of ICP show conflicting results which likely reflect the uncertainty in the estimated ICP. This uncertainty calls for invasive ICP assessment for investigation of pathophysiological importance of ICP and TLCPD in glaucoma.

We have found six studies where invasive ICP measurement was performed on patients with glaucoma or ocular hypertension (Table 1) [7–9, 82–84]. Berdahl et al. performed a retrospective analysis on a large dataset with patients that had undergone LP due to different neurological reasons [7, 8•]. In their initial 10-year chart review, they found lower ICP in glaucomatous patients than in the selected controls [7]. The chart review was extended to approximately 20 years. The analysis of the glaucoma patients included a subgroup of NTG as well as a group of patients at risk of developing glaucoma, i.e., ocular hypertension (OH), as sampled and compared with age-matched non-glaucomatous controls [8•]. As expected, the results showed lower ICP in NTG and HTG, which confirmed their earlier results. Interestingly, patients with OH were found to have elevated ICP, which could indicate a protective marker for development of glaucoma. TLCPD was higher in POAG and NTG than in the control group [8•].

**Table 1:** Summary of results from studies that compare intracranial pressure in patients with glaucoma/ocular hypertension and controls

		NTG		HTG		POAG		OH		Controls		Patients vs controls
Author (year)	Design	*N*	ICP	*N*	ICP	*N*	ICP	*N*	ICP	*N*	ICP	*P* value
Berdahl 2008 [7]	Retrospective	–	–	–	–	28	9.2 (± 2.9)	–	–	49	13.0 (± 4.2)	< 0.00005
Berdahl 2008 [8•]	Retrospective	11	9.3 (± 3.2)	–	–	57*	9.6 (± 3.1)	27	13.2 (3.8)	66 (POAG)	12.7 (± 3.9)	< 0.0001 (POAG)
										39 (OH)	11.5 (± 3.3)	
												< 0.01 (NTG)
												ns (OH)
Ren 2010 [9]	Prospective	14	9.5 (± 2.2)	29	11.7 (± 2.7)	–	–	–	–	71	12.9 (± 1.9)	< 0.001 (NTG)
												< 0.001 (HTG)
Ren 2011 [10]	Prospective	–	–	–	–	–	–	17	16.0 (± 2.5)	71	12.9 (± 1.9)	< 0.001
Pircher [79]	Retrospective	38	11.6 (± 3.7)	–	–	–	–	–	–	–	–	na
Lindén [82]	Prospective	13	10.3 (± 2.7)	–	–	–	–	–	–	51	11.3 (± 2.2)	ns

Measurements of ICP are expressed in mmHg

*NTG* normal-tension glaucoma, *HTG* high-tension glaucoma, *OH* ocular hypertension, *ICP* intracranial pressure

*including NTG subgroup

Ren et al. performed prospective studies on ICP and TLCPD in Chinese patients with OAG and OH. When 43 patients with OAG where compared with 71 non-glaucomatous controls, ICP was significantly lower in patients with NTG than HTG or controls. Accordingly, the TLCPD was elevated in NTG patients [9]. When OH patients were compared with the same control group, ICP was significantly higher [84].

In a recent non-controlled retrospective study on 38 NTG patients, Pircher et al. found an ICP of 11.6 mmHg and TLCPD of 3.0 mmHg [83]. Thus, the study did not confirm previous findings of low ICP and high TLCPD [8•, 9].

The only study on glaucoma patients with direct measurement of ICP and simultaneous measurements of IOP and ICP in different postures is a recent investigation by Lindén et al. (2017). In that prospective study on NTG patients compared with healthy age-matched controls, all NTG patients underwent washout of IOP-lowering medication. The findings showed no significant difference between the groups with respect to ICP or TLCPD [82], so even for invasive ICP studies, there are conflicting results. however, these are all studies with few subjects and a large multicenter study is needed.

Although the literature cannot strictly confirm a reduced ICP in NTG, this does not exclude the possibility that an increased TLCPD is a part of the pathophysiology. There are findings of an increased risk of NTG in shunt-treated normal pressure hydrocephalus patients supporting that the ICP lowering effect of the shunt is correlated to development of NTG [85]. Additionally, there are mechanisms of transfer of the ICP pressure to the ONSAS and to the retrolaminar tissue that need to be investigated. Thus, when assessing TLCPD, there are several hurdles and limitations that need to be overcome in order to get reliable measurements of IOP, ICP, and ultimately the retrolaminar pressure.

## Limitations in Studies on TLCPD

Firstly, the site of measurement of IOP and ICP is not the same. IOP is indirectly measured at the cornea and ICP in the current clinical studies was measured in the spinal canal with a direct measurement through LP. In both IOP and ICP measurements, an assumption is often made that the pressure at LC is the same as at the site of measurement. The finding of Lenfelt et al. [35] that lumbar CSF pressure is in agreement with intra-parenchymal ICP supports this. However, that assumption is criticized by others who have pointed out that the data that support equal CSF flow throughout the CSF regions are questionable [83]. Furthermore, Killer et al. [86] have described compartmentation of the subarachnoid space (SAS) surrounding the optic nerve, which may affect the relationship between ICP and the retrolaminar pressure. Studies of local ICP in the ONSAS are scarce and limited to animal models or cadaver eyes that are inconclusive [27, 28], i.e., we do not really know the retrolaminar pressure in humans.

Secondly, body position is crucial in comparison of IOP and ICP. It is well known that both IOP [87] and ICP [88] are affected by posture. The pressure increases in the supine position as compared to the upright position but to a different extent [57]. In most studies, TLCPD is calculated with measurements of IOP performed with the subject in a sitting position and ICP in supine position. Thus, that TLCPD is significantly affected depending on the posture is not accounted for [57].

Thirdly, the time point of measurement is important. IOP is known to fluctuate depending on the time of the day [89], but less is known about circadian fluctuations of ICP that are independent of posture [22]. This needs to be taken into account in study design where simultaneous measurement of IOP and ICP is preferable.

Fourthly, drugs affecting IOP or ICP need to be considered as they may skew the comparison. Especially in studies on glaucoma and TLCPD, IOP-lowering treatment is often continued during the study, and no washout period is performed. This confounds the results as the IOP will be lower due to treatment.

## Effects of Posture on ICP and IOP

As pointed out previously, a common limitation in studies on TLCPD is that ICP and IOP are not measured in the same posture [7, 8•, 9, 83, 84]. This important bias makes calculation of TLCPD questionable. Recent studies with simultaneous measurements of ICP and IOP in different postures have described the physiology of postural changes on both pressures in healthy subjects [57] as well as in patients with NTG [82].

An important aspect is the 24-h postural influence on TLCPD in terms of lying down or standing/sitting upright. With the assumption of a communicating system between the intracranial and ONSAS CSF, it has been shown that TLCPD can differ from 12 mmHg in supine to 20 mmHg in upright posture. Assuming that we lie down one third of the time, the 24-h TLCPD average would be approximately 17 mmHg [57]. Thus, if there is free communication between the ICP regions and the subarachnoid space surrounding the optic nerve behind the LC, then the LC is exposed to great variations in pressure gradient. Given these large differences in TLCPD due to postural changes, the reported differences in ICP between patients with glaucoma and controls seem less clinically relevant.

**Fig. 1:**
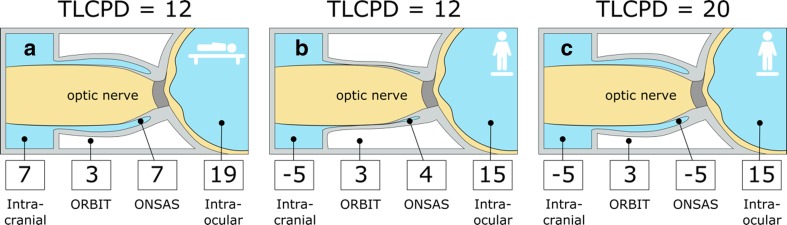
Translamina cribrosa pressure difference (TLCPD) dependency on possible occlusion of the optic nerve sheath and its potential importance in normal-tension glaucoma (NTG). The illustrations show four compartments with corresponding pressures, i.e., intracranial pressure (ICP) and intraocular pressure (IOP) at the level of the eye [87], pressure in the orbit [89] as well as hypothetical pressure retrolaminarly in the optic nerve subarachnoid space (ONSAS). **a** In supine position, TLCPD is 12 mmHg. **b** In upright position and when ICP decreases substantially, an occlusion mechanism of the optic nerve sheath may preserve higher pressure in ONSAS as it becomes equal to the orbital pressure and thus keep TLCPD stabile at 12 mmHg. **c** If this occlusion mechanism is deficient and an open fluid communication is present, which we hypothesize in NTG, then the retrolaminar pressure would be the same as the ICP, resulting in an elevated and potentially harmful TLCPD in upright position

It is evident that posture plays a key role in the assessment of TLCPD since ICP is much more affected by postural changes than IOP, and that results in large differences in TLCPD during the 24-h cycle. This indicates that there is another control function in the intraocular system compared with the intracranial one. Results from an animal study [27] have indicated that when ICP is reduced below the surrounding intraorbital pressure, an occlusion of the optic nerve sheath occurs around the optic nerve as part of normal physiology. This occlusion mechanism might then protect the axons from the low ICP when the subject has an upright posture [16]. An attractive hypothesis for a possible role of TLCPD in glaucoma would therefore be a disturbance in this protective mechanism. A pathologically stiff optic nerve sheath or a partial obstruction in the ONSAS might cause deficient occlusion and thus result in abnormally high TLCPD at the LC as the protection from low ICP would not be in place. This hypothesis is illustrated in Fig. 1.

There are other theories related to the communication between all spaces of the CSF system. Instead of a deficient occlusion mechanism (Fig. 1), Morgan et al. hypothesized that low orbital pressure or a less elastic pia mater might help convey lower pressures to the retrolaminar tissue resulting in higher TLCPD [14•]. Killer et al. reported compartmentation in the ONSAS in NTG patients [86], which possibly causes pressure in the comparted ONSAS to be different than in other CSF regions [86, 90]. In contrast to the hypothesis we propose in this paper (Fig. 1), i.e., a non-occluding optic nerve sheath in NTG, they assume a constant occlusion/restriction, which causes an impaired circulation of CSF in the compartmented ONSAS, which leads to stasis of CSF as well as reduced clearance of toxins, both being detrimental to the axons.

Another important aspect is whether difference in ICP or TLCPD could be considered clinically relevant. In the case of NTG, a 30% reduction of IOP compared with baseline has been shown to reduce the progression of the disease [91, 92]. This would translate into a pressure reduction of 5 mmHg in patients presenting with IOP of 16 mmHg [91, 92]. In previously mentioned studies, which measured ICP directly in NTG patients, ICP was shown to range between 9.3 and 11.6 mmHg [8, 9, 82, 83]. When a non-glaucomatous but not always entirely healthy, control group was available, the ICP was shown to range between 11.3 and 12.9 mmHg. It is a matter of debate if this difference in ICP between the NTG and controls is clinically relevant, especially given the much larger difference, which occurs due to postural changes.

## Conclusion and Future Perspectives

A pathological TLCPD is an attractive hypothesis in pathophysiology theories of certain diseases. For IIH, ICP is elevated and for SANS, it is suspected to be elevated and thus reduced or reversed TLCPD is excepted as part of these diseases. For glaucoma and in particular NTG, it is more controversial. The present literature review revealed a large number of review papers, which indicates a great interest in the theory, while only a few research studies in humans have been reported. Initial studies supported the hypothesis while recent findings indicate that simply a reduced ICP might not be a distinct characteristic of the NTG group. Importantly, this does not exclude that elevated TLCPD due to an abnormally low retrolaminar pressure caused by dysfunctional optic nerve sheath mechanisms may be a factor. We note that this is still an emerging field, and interdisciplinary collaboration is required between ophthalmology and neurology expertise and that methodological developments of the current approaches are needed. From the identification of potential postural effects on the CSF communication between the intracranial and retrolaminar space, future challenges include to further study if an occlusion mechanism of the optic nerve sheath exists in the human intraorbital space and if so, investigate elevated TLCPD in relation to failure in this mechanism as a potential pathophysiological component in NTG patients.
